# Protein Preconcentration Using Nanofractures Generated by Nanoparticle-Assisted Electric Breakdown at Junction Gaps

**DOI:** 10.1371/journal.pone.0102050

**Published:** 2014-07-15

**Authors:** Chun-Ping Jen, Tamara G. Amstislavskaya, Chen-Chi Kuo, Yu-Hung Chen

**Affiliations:** 1 Department of Mechanical Engineering and Advanced Institute of Manufacturing with High-Tech Innovations, National Chung Cheng University, Chia Yi, Taiwan, R.O.C.; 2 Institute of Cytology and Genetics, Siberian Branch of Russian Academy of Sciences, Novosibirsk, Russia; 3 Department of Medicine, National Cheng-Kung University, Tainan, Taiwan, R.O.C.; 4 Department of Biochemistry and Molecular Biology, National Cheng-Kung University, Tainan, Taiwan, R.O.C.; Northeastern University, United States of America

## Abstract

Sample preconcentration is an important step that increases the accuracy of subsequent detection, especially for samples with extremely low concentrations. Due to the overlapping of electrical double layers in the nanofluidic channel, the concentration polarization effect can be generated by applying an electric field. Therefore, a nonlinear electrokinetic flow is induced, which results in the fast accumulation of proteins in front of the induced ionic depletion zone, the so-called exclusion-enrichment effect. Nanofractures were created in this work to preconcentrate proteins via the exclusion-enrichment effect. The protein sample was driven by electroosmotic flow and accumulated at a specific location. The preconcentration chip for proteins was fabricated using simple standard soft lithography with a polydimethylsiloxane replica. Nanofractures were formed by utilizing nanoparticle-assisted electric breakdown. The proposed method for nanofracture formation that utilizes nanoparticle deposition at the junction gap between microchannels greatly decreases the required electric breakdown voltage. The experimental results indicate that a protein sample with an extremely low concentration of 1 nM was concentrated to 1.5×10^4^-fold in 60 min using the proposed chip.

## Introduction

The development of microfluidic platforms for analyzing chemical and biological samples has increased dramatically in the past decade. Sample preconcentration is an important step that increases the accuracy of subsequent detection, especially for samples with extremely low concentrations. Several strategies, including field-amplified sample stacking (FASS) [1], [2], isotachophoresis (ITP) [3], isoelectric focusing (IEF) [4], temperature gradient focusing (TGF) [5], nanofilters [6], and nanoporous membrane/nanochannel techniques [7], [8], [9], [10], have been employed to preconcentrate proteins with low concentrations [11]. These procedures are complicated and at least two kinds of buffer solution are required for FASS, ITP, and IEF. TGF is based on the fact that the electrophoretic velocity of the analyte changes as a function of temperature. Therefore, precise temperature control is necessary for TGF. A method that uses the filtering effect to stack analytes at one side of a nanoporous membrane or nanochannel with pore sizes or channel dimensions smaller than that of analytes has been proposed [6]. Analytes can also be concentrated by utilizing the exclusion-enrichment effect [12] at the nanopores or nanochannels. Due to the overlapping of electrical double layers in the nanofludic channel, an inherent ion-permselectivity allows counterions to pass through the nanochannel but repels co-ions out of the nanochannel. Under the action of an electric field, the concentration polarization effect generates the ionic depletion effect at the anodic side of the nanochannel. Therefore, a nonlinear electrokinetic flow is induced, which results in the fast accumulation of proteins in front of the induced ionic depletion zone, the so-called exclusion-enrichment effect [12]. The major benefit of this method is that simple buffer systems can be adopted [13]. The key technique for applying the exclusion-enrichment effect is creating nanofluidic channels. Several approaches are currently available for the fabrication of nanochannels/nanopores [14]. Wang et al. [7] proposed a microdevice with nanofluidic channels fabricated with standard photolithography and etching techniques to generate an extended space charge region for electrokinetically collecting and trapping proteins with concentration factors as high as 10^6^–10^8^. However, this fabrication process, although highly accurate [15], utilizes reactive ion etching (RIE) and is thus time-consuming and costly [11]. The use of porous membrane techniques is attractive due to the fact that commercially available membranes can be easily integrated onto microchips. Polydimethylsiloxane (PDMS) microdevices integrated with polycarbonate track etched (PCTE) membranes with 10-nm nanopores [6] have been shown to have an accumulation factor of 10^5^–10^6^. A highly ion-conductive charge-selective polymer, poly-AMPS (2-acrylamido-2-methyl-1-propanesulfonic acid), has been employed for a microfluidic sample preconcentration system [8]. The achieved concentration factor of a tetramethylrhodamine isothiocyanate (TRITC)-tagged bovine albumin sample was 10^3^ in 20 min. Furthermore, Nafion resin, a highly porous ion-selective material, has been widely integrated with PDMS/glass-based microfluidic chips. A PDMS microfluidic chip bonded on the top of a glass substrate with a surface-pattern printed on a submicron-thick Nafion film has been proposed for multiplexed proteomic sample preconcentration, achieving a concentration factor of as high as 10^4^ in 5 min [8]. Kim and Han [9] extended their previous work, mentioned above, and developed a simple method that integrates polymeric nanoporous junctions into a PDMS microchip. The PDMS gap created by mechanical cutting was infiltrated with the Nafion polymer solution and self-sealed due to its flexibility. The preconcentration of *β*-phycoerythrin proteins in large channels (dimensions: 1000 µm (width)×100 µm (depth)) was achieved with a concentration factor of up to 10^4^. Moreover, a massive array of 128 parallel nanofluidic concentration microdevices with Nafion nanoporous junctions for high-throughput biomolecule detection has been fabricated and shown to greatly increase the dynamic range of immunoassays [16]. Nanoporous membranes are not easily embedded into microchips without leakage of the liquid. Although the use of photopolymerization might overcome this problem, a complicated optical setup and careful operation are needed to accomplish the process [17]. A simpler technique for fabricating nanochannels or nanofractures, which makes use of the junction-gap electric breakdown between two PDMS microchannels [18], has been proposed to address this issue. Nanogaps form between the PDMS microchannels when a high voltage is applied. A direct-current (DC) voltage of 1000 V was applied between microchannels 40 µm in width; the corresponding electric field of 25 V/µm was slightly greater than the dielectric strength of PDMS (21 V/µm), creating a nanogap with a depth of approximately 80 nm. A concentration factor of as high as 10^4^ within 1 h was demonstrated by Lee et al. [18]. Spontaneously formed nanochannels underneath the PDMS layer were reversibly and weakly bonded to a glass substrate in another study [19]. A concentration factor of 10^3^ to 10^6^ was achieved in 30 min on a microchip with chevron-shaped microchannels in a mirror-image orientation. However, the use of reversible bonding between PDMS and a glass substrate is less robust than the permanent bonding process obtained using oxygen-plasma treatment. A microchip with two printed V-shaped microchannels in a mirror-image orientation, separated by a 100-µm gap, was presented by Yu et al. [17]. Nanofractures were then formed by electric breakdown under a high electric field, with resulting concentration factors of 10^3^–10^5^ for proteins. Compared to the porous membrane-based technique described previously, the nanogaps were fabricated without the use of special reagents or materials; instead, junction-gap electric breakdown was employed. However, the required voltage to initiate electric breakdown is high and the cross-sectional areas of the nanogaps are smaller than those of a porous membrane [11]. The exclusion-enrichment effect in a nanofluidic channel is employed to preconcentrate proteins in the present investigation. The protein sample is driven by an electroosmotic flow and accumulates at a specific location. The proposed nanofluidic chip for the preconcentration of proteins is fabricated using simple standard soft lithography with a PDMS replica. Nanofractures are formed using nanoparticle-assisted electric breakdown. The proposed method for nanofracture formation utilizes nanoparticle deposition at the junction gap between microchannels to reduce the required electric breakdown voltage.

## Materials and Methods

### Design and fabrication of microchips

**Figure 1:**
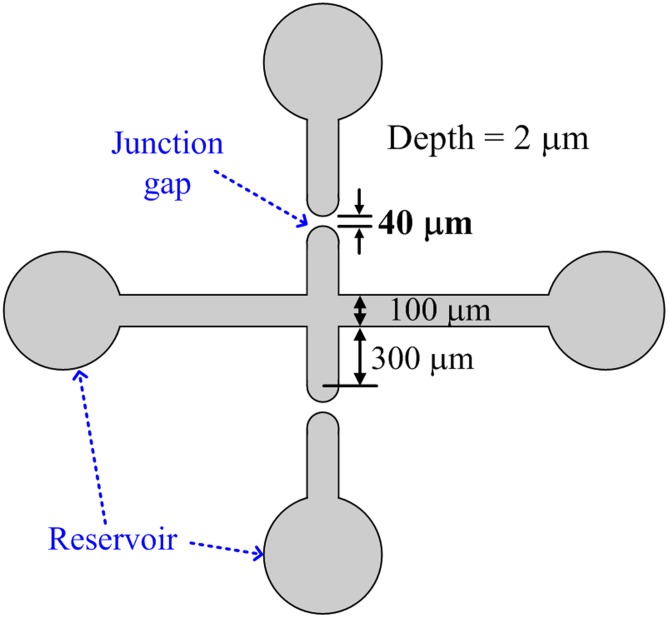
Layout and dimensions of preconcentration chip.

**Figure 2:**
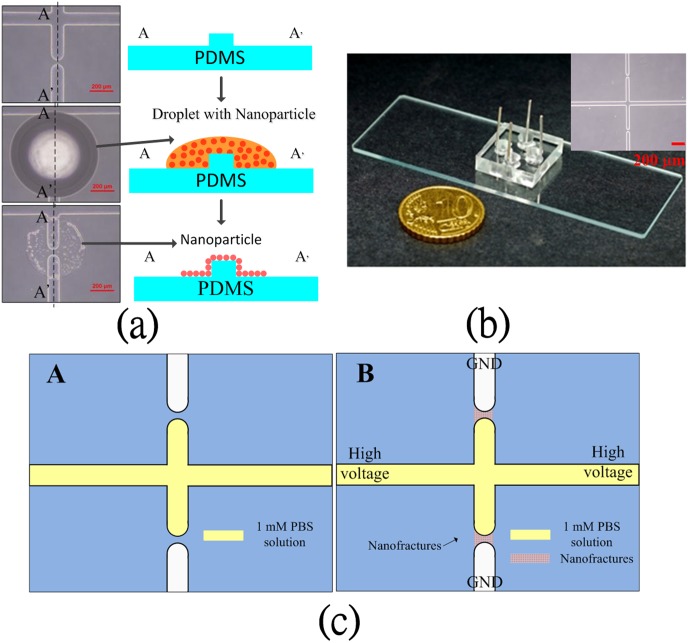
(a) Schematic diagram of depositing nanoparticles to assist electric breakdown on the chip; (b) image of fabricated chip (optical microscopy image in inset) and (c) illustrations of nanofracture formation for protein preconcentration.

PDMS, a biocompatible and transparent material, was adopted for the micro/nanochannels in the chip for protein preconcentration. The layout and dimensions of the preconcentration chip are shown in Fig. 1. Two junction gaps were designed along the main microchannel for the formation of nanofractures via electric breakdown. The depth and width of the main microchannel are 2 µm and 100 µm, respectively. The width of the junction gap is 40 µm. The mold master was fabricated by spinning S1818 (Rohm and Haas Electronic Materials LLC, Philadelphia, PA, USA) on a silicon wafer (around 2 µm thick) to define the microchannel. A 5∶1 weight mixture of PDMS prepolymer and curing agent (Sylgard-184 Silicone Elastomer Kit, Dow Corning, Midland, MI, USA) was poured and cured on the mold master to replicate the microchannel. After the PDMS replica had been peeled off, the inlet and outlet ports were made by a puncher, and a 100-nL droplet of liquid containing gold nanoparticles was dropped onto the junction gaps to reduce the required breakdown voltage. The procedure of depositing nanoparticles on the junction gaps is illustrated in Fig. 2a. Gold colloids were prepared based on a procedure from the Natan method [20]. A solution of tetrachloroauric acid was synthesized by dissolving 39.37 mg of HAuCl_4_·3H_2_O in 100 mL distilled deionized water. After the solution was heated to a boil, 10 mL of 38.8 mM aqueous sodium citrate solution was added to it under vigorous stirring to form a claret solution. The mean diameter of Au nanoparticles was 13.7±0.8 nm. The original particle concentration was approximate 20 nM; however, the droplet containing nanoparticles was diluted to 0.02, 0.2, and 2 nM, respectively, before being dropped onto the nanogaps. After the 100-nL droplet on the junction gap had evaporated, the PDMS replica was bonded to the glass substrate using oxygen-plasma treatment in an O_2_ plasma cleaner (PDC-32G, Harrick Plasma Corp., Ithaca, NY, USA). After oxygen plasma treatment, the hydrophobic –OSi(CH_3_)_2_O– groups converts to hydrophilic –O_n_Si(OH)_4-n_. Indeed, this hydrophilic surface is unstable and will recover to the original –OSi(CH_3_)_2_O– groups within an hour. However, immediate contact of the hydrophilic PDMS surface to glass forms irreversible covalent (Si–O–Si) bonds through condensation reaction [21], [22]. Therefore, the surface chemistry of the proposed microchip between PDMS and glass is through covalent binding rather than charges. Four electrodes were then inserted into the reservoirs to apply the required voltage in the experiments. The fabricated chip and its optical microscopy image are shown in Fig. 2b and its inset, respectively.

### Formation of nanofractures and protein preconcentration

Nanofractures were created between microchannels via junction gap breakdown. Phosphate-buffered saline (PBS; 1 mM, pH 7.4) solution was used for the buffer system in the present study. A DC voltage was applied to the two anodic side reservoirs while the other reservoirs were grounded, as shown in Fig. 2c, to initiate electric breakdown and thus nanofracture formation. After the nanofractures were created, their electric characteristics were measured by an LCR meter to determine the nanofracture formation criteria. Fluorescein isothiocyanate (FITC)-labeled bovine serum albumin (BSA) (Sigma-Aldrich, St. Louis, MO, USA) diluted in 1 mM PBS buffer at concentrations of 1 nM and 100 nM was filled into the microchannel by capillary force to demonstrate the on-chip preconcentration of proteins.

### Apparatus

A high-voltage power supply (Series 225, Bertan High Voltage Corp., Hicksville, NY, USA) was employed to provide the required voltages for both junction gap breakdown and protein preconcentration. After the nanofractures were created, the resistance difference between the junction gaps before and after the application of a DC voltage for electric breakdown was measured under various frequencies using an LCR meter (4620A, Wayne Kerr, Woburn, MA, USA). The preconcentration of proteins was observed and recorded using an inverted fluorescence microscope (CKX41, Olympus, Tokyo, Japan) with a mounted CCD camera (DP71, Olympus, Tokyo, Japan) and connected to a computer with Olympus DP Controller image software. Quantification of the fluorescent intensities emitted by enriched FITC-labeled BSA was performed using NIH ImageJ software (National Institutes of Health, Bethesda, Maryland, USA), which has the ability to assess the density of each pixel.

## Results and Discussion

**Figure 3:**
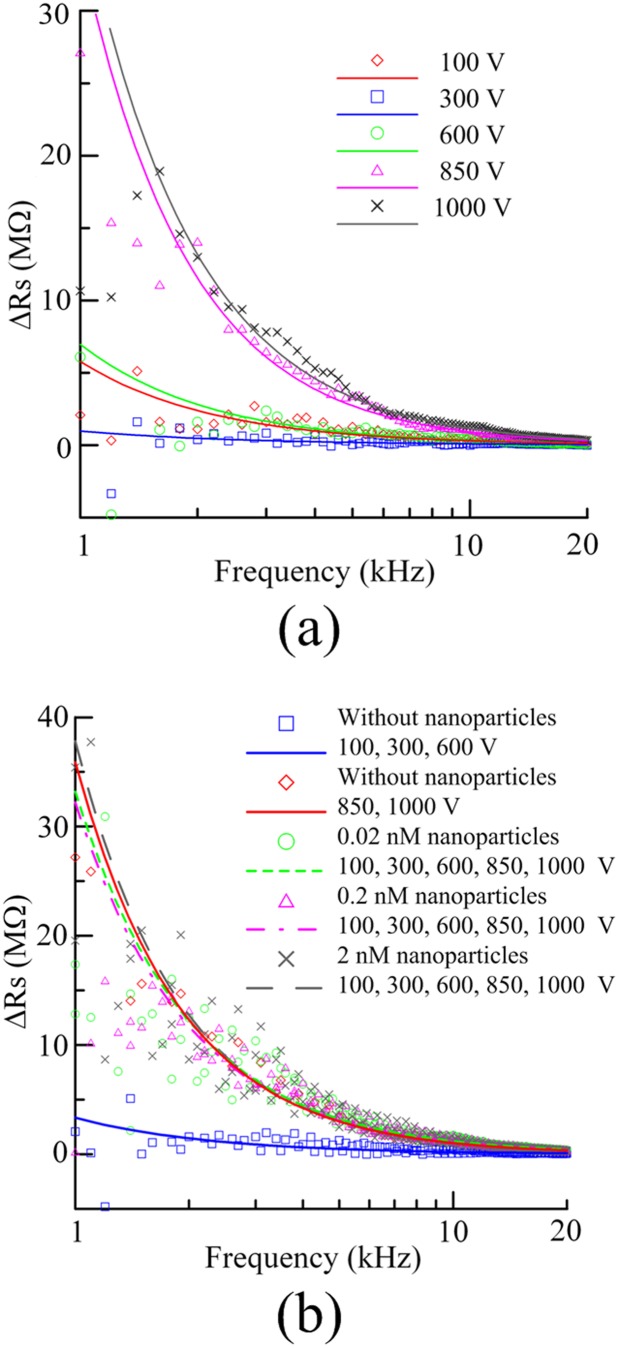
Differences in resistances between the junction gap before and after nanofracture formation measured as a function of frequency (a) without nanoparticle deposition and (b) with deposition of 0.02, 0.2, and 2 nM of nanoparticles.

The conductivity of a rectangular-shaped nanochannel was previously measured by Stein et al. [23], who found that the electrical conductance of channels saturates at a value that is independent of both the salt concentration and the channel height under low-salt conditions. DC current measurements as a function of applied voltage (I–V curve) were performed to investigate the formation and dimensions of the nanogap using a relatively high concentration of KCl electrolyte (10^−1^ M) in a previous study [18]. However, since the gold nanoparticles were deposited onto the surface of the protein preconcentrator manually in the present study, the amount and distribution of nanoparticles was not well controlled and thus the variations in DC current measurements between chips were significant. After nanofractures between microchannels were created after junction gap breakdown under various applied voltages for 5 min, an LCR meter was employed to measure the resistance spectrum under a relative low concentration of PBS (1 mM) to confirm the formation of nanofractures only. Dimensions of the nanofractures are not investigated herein. Differences in resistances between the junction gap before and after nanofracture formation measured as a function of frequency without nanoparticle deposition and with the deposition of 0.02, 0.2, and 2 nM of nanoparticles are shown in Fig. 3. The effective resistances of nanofractures both with and without nanoparticle deposition increase with frequency due to the skin effect, which is due to the fact that the internal inductance of a conductor is highest at the center and lowest at the edges [24]. Differences in resistances between the junction gap before and after nanofracture formation thus decrease with increasing frequency, as depicted in Fig. 3. For the cases without nanoparticle deposition, various DC voltages were applied to initiate the electric breakdown, as shown in Fig. 3a. The resistance spectra obtained for voltages larger than 840 V (the dielectric strength of PDMS) are different from those obtained for voltages less than 840 V. When the applied voltages were insufficient for creating nanofractures, differences in resistances between the junction gap before and after the application of voltages (100, 300, and 600 V) were small and nanofractures did not form. Nanofractures were created when the applied voltage was larger than 840 V. Figure 3b shows the resistance spectra for cases with the deposition of nanoparticles. The results indicate that the resistance spectra for all cases with nanoparticle deposition are similar to those obtained for applied voltages larger than 840 V without nanoparticle deposition. Hence, nanofractures were created with applied voltages as low as 100 V when a droplet of nanoparticles with a concentration of 0.02 nM was deposited onto the junction gaps.

**Figure 4:**
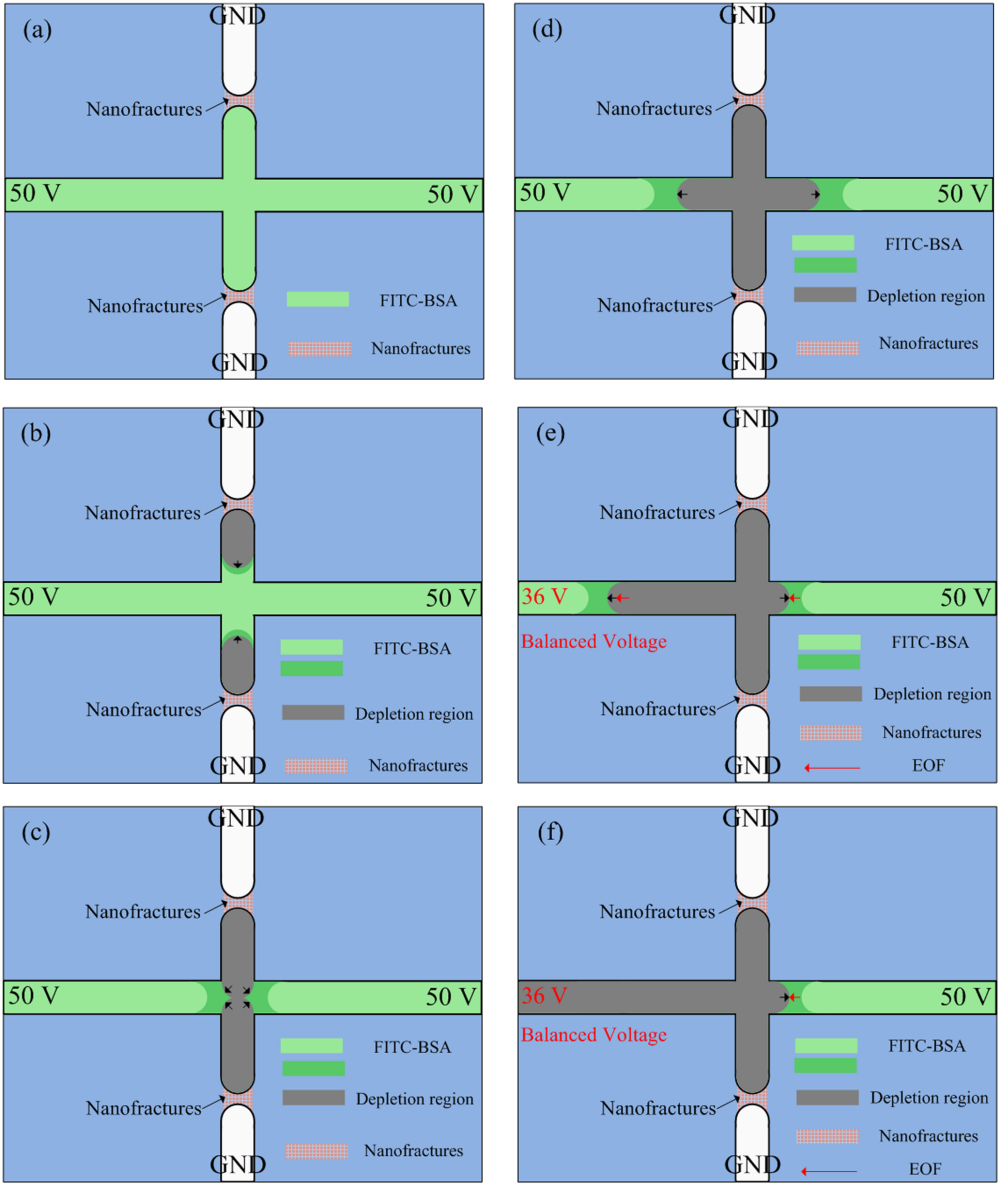
Schematic diagram of operations of electrokinetic protein preconcentration.

**Figure 5:**
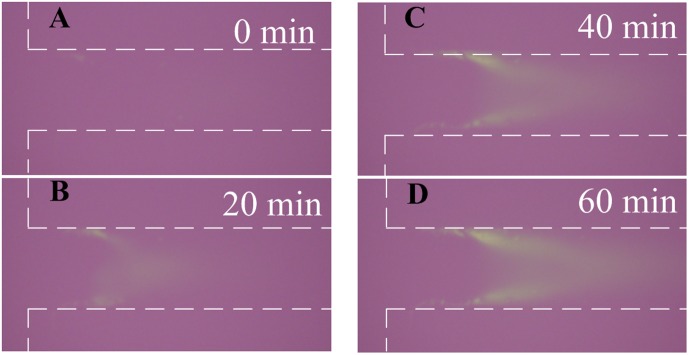
Fluorescence images of 100 nM FITC labeled BSA in 1 mM PBS buffer solution (pH 7.4) taken at various time points. Chip with 2 nM of gold nanoparticles at the junction gaps had a DC voltage of 300 V applied to it for 5 min to create nanofractures.

**Figure 6:**
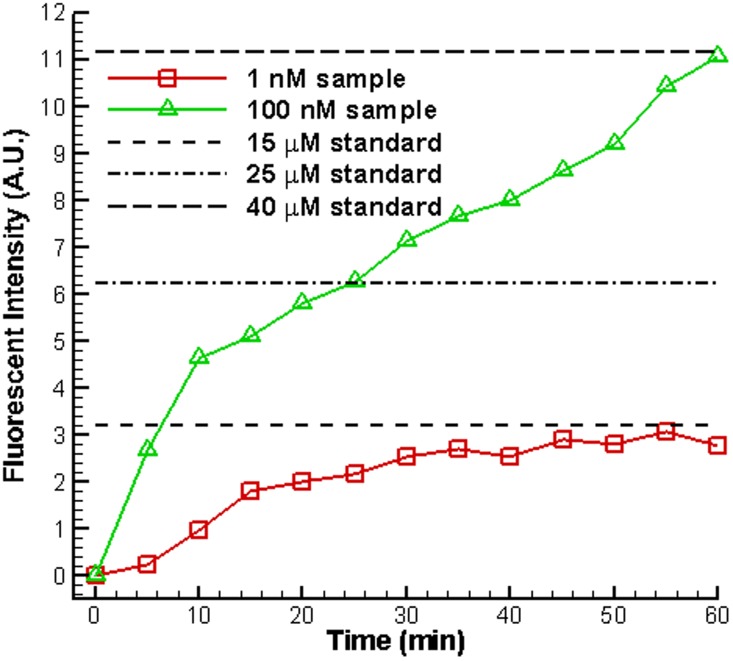
Concentration performance initial protein concentrations of 1 nM and 100 nM. Chip with 2 nM of gold nanoparticles at the junction gaps had a DC voltage of 300 V applied to it for 5 min to create nanofractures.

After nanofractures were generated, FITC-labeled BSA diluted in 1 mM PBS buffer at concentrations of 1 nM and 100 nM was filled into the microchannel by capillary force to perform the on-chip preconcentration of proteins. A schematic diagram of the operations of the electrokinetic protein preconcentration is illustrated in Fig. 4. The depletion regions in the main vertical channel elongate when a DC voltage of 50 V is applied onto the two anodic side reservoirs while the other reservoirs are grounded, as shown in Figs. 4a and 4b. The elongating depletion regions from the top and bottom junction gaps merge with each other and extend to the main horizontal channel, as depicted in Figs. 4c and 4d. Then, a bias voltage of 36 V is set at the left anodic side to induce electroosmotic flow (EOF), as shown in Fig. 4e. The proteins accumulate and become stacked in the region near the intersection of the microchannels, as illustrated in Fig. 4f. However, although nanofractures formed for all cases with the deposition of nanoparticles, the depletion force generated by the nanofractures might not large enough for concentrating proteins. A droplet with nanoparticles diluted to 2 nM for deposition and 300 V for electric breakdown were chosen as suitable parameters for obtaining reliable results of protein preconcentration after all the parameters in Fig. 3 had been tested [25]. Fluorescence images of 100 nM FITC-labeled BSA in 1 mM PBS buffer solution taken at various time points are shown in Fig. 5. The results reveal that both the concentration of BSA and the size of the chevron-shaped preconcentration area increased with time. The concentration of collected BSA protein obtained from fluorescence intensity was quantified and averaged over a rectangular window using NIH ImageJ software. The concentration performance for initial protein concentrations of 1 nM and 100 nM is plotted in Fig. 6. To estimate the final concentration, the fluorescence intensity of the standard sample solutions (15, 25, and 40 µM) was also measured and illustrated in this figure. Experimental results indicate that the protein sample with a concentration of 100 nM became concentrated to 40 µM in 60 min (about 400-fold the initial concentration). Moreover, the protein sample with an extremely low concentration of 1 nM was concentrated to 1.5×10^4^-fold in 60 min.

## Conclusion

A method for the formation of nanofractures that utilizes nanoparticle deposition at the junction gap between microchannels was proposed to reduce the required electric breakdown voltage. A 100-nL droplet of liquid containing gold nanoparticles was dropped onto the junction gaps before the voltage for electric breakdown was applied. After nanofractures were formed using the nanoparticle-assisted electric breakdown between microchannels under various applied voltages, the resistance spectrum was measured by an LCR. The measured results reveal that nanofractures were created for applied voltages of as low as 100 V when a droplet of nanoparticles with a concentration of 0.02 nM was deposited onto the junction gaps. However, although nanofractures were formed for all cases with the deposition of nanoparticles, the depletion force generated by the nanofractures might not large enough for concentrating proteins. A droplet with nanoparticles diluted to 2 nM for deposition and 300 V for electric breakdown were chosen as suitable parameters for providing reliable results of protein preconcentration. Experimental results reveal that a protein sample with a concentration of 100 nM became concentrated to 400-fold the initial concentration. Moreover, a protein sample with an extremely low initial concentration of 1 nM was concentrated to 1.5×10^4^-fold in 60 min. The concentration factor of the proposed design is comparable to that of existing devices; however, the required voltage is only 36% of that without nanoparticles deposition. The electrokinetic preconcentration of proteins using nanofractures generated by nanoparticle-assisted electric breakdown at the junction gaps was demonstrated.
